# Categorizer: a tool to categorize genes into user-defined biological groups based on semantic similarity

**DOI:** 10.1186/1471-2164-15-1091

**Published:** 2014-12-11

**Authors:** Dokyun Na, Hyungbin Son, Jörg Gsponer

**Affiliations:** Department of Biochemistry and Molecular Biology, Centre for High-throughput Biology, University of British Columbia, 2125 East Mall, Vancouver, BC V6T 1Z4 Canada; School of Integrative Engineering, Chung-Ang University, 84 Heukseok-ro, Dongjak-gu, Seoul, 156-756 Republic of Korea

**Keywords:** Gene ontology, Categorization, Enrichment analysis, Semantic similarity, Neurodegenerative diseases

## Abstract

**Background:**

Communalities between large sets of genes obtained from high-throughput experiments are often identified by searching for enrichments of genes with the same Gene Ontology (GO) annotations. The GO analysis tools used for these enrichment analyses assume that GO terms are independent and the semantic distances between all parent–child terms are identical, which is not true in a biological sense. In addition these tools output lists of often redundant or too specific GO terms, which are difficult to interpret in the context of the biological question investigated by the user. Therefore, there is a demand for a robust and reliable method for gene categorization and enrichment analysis.

**Results:**

We have developed Categorizer, a tool that classifies genes into user-defined groups (categories) and calculates *p*-values for the enrichment of the categories. Categorizer identifies the biologically best-fit category for each gene by taking advantage of a specialized semantic similarity measure for GO terms. We demonstrate that Categorizer provides improved categorization and enrichment results of genetic modifiers of Huntington’s disease compared to a classical GO Slim-based approach or categorizations using other semantic similarity measures.

**Conclusion:**

Categorizer enables more accurate categorizations of genes than currently available methods. This new tool will help experimental and computational biologists analyzing genomic and proteomic data according to their specific needs in a more reliable manner.

## Background

During the last decade, high-throughput technologies have allowed scientists to collect large sets of genomic and proteomic data. These data sets are then often screened for groups of genes that are over-represented or depleted when compared to a reference set or the entire genome/proteome of a specific organism. Therefore, great efforts have been made to develop computational methods to translate the flourishing raw data into meaningful biological knowledge.

Gene Ontology (GO) is a dictionary of controlled biological vocabularies to annotate genes at different levels of granularity [1]. The GO dictionary can be envisioned as a graph that has, in a first approximation, the architecture of an upside down tree in which connected nodes, i.e., related GO terms, have a parent–child relationship and all nodes can be connected back to the three root nodes (*biological process*, *molecular function* and *cellular component*). This well-structured knowledge has been utilized to identify specific biological processes or functions enriched within sets of genes. There are many tools that can carry out this task: David, FuncAssociate, BiNGO, etc. [2–5]. These tools output lists of all individual GO terms that are significantly enriched in the analyzed data set. However, listed GO terms often refer to the same biological process. In addition, many GO terms are highly specific and difficult to interpret in the larger biological context that is investigated. As the research of most scientists is focused on a specific area, scientists are often less interested in the enrichment of specific GO terms in a set of genes but more in the enrichment of all GO terms that are associated with their area of interest. In order to reach this goal, researchers define categories of interest and manually assign genes into one of these categories according to their GO annotations [6, 7]. This is a laborious process and categorization results may differ from person to person.

**Figure 1:**
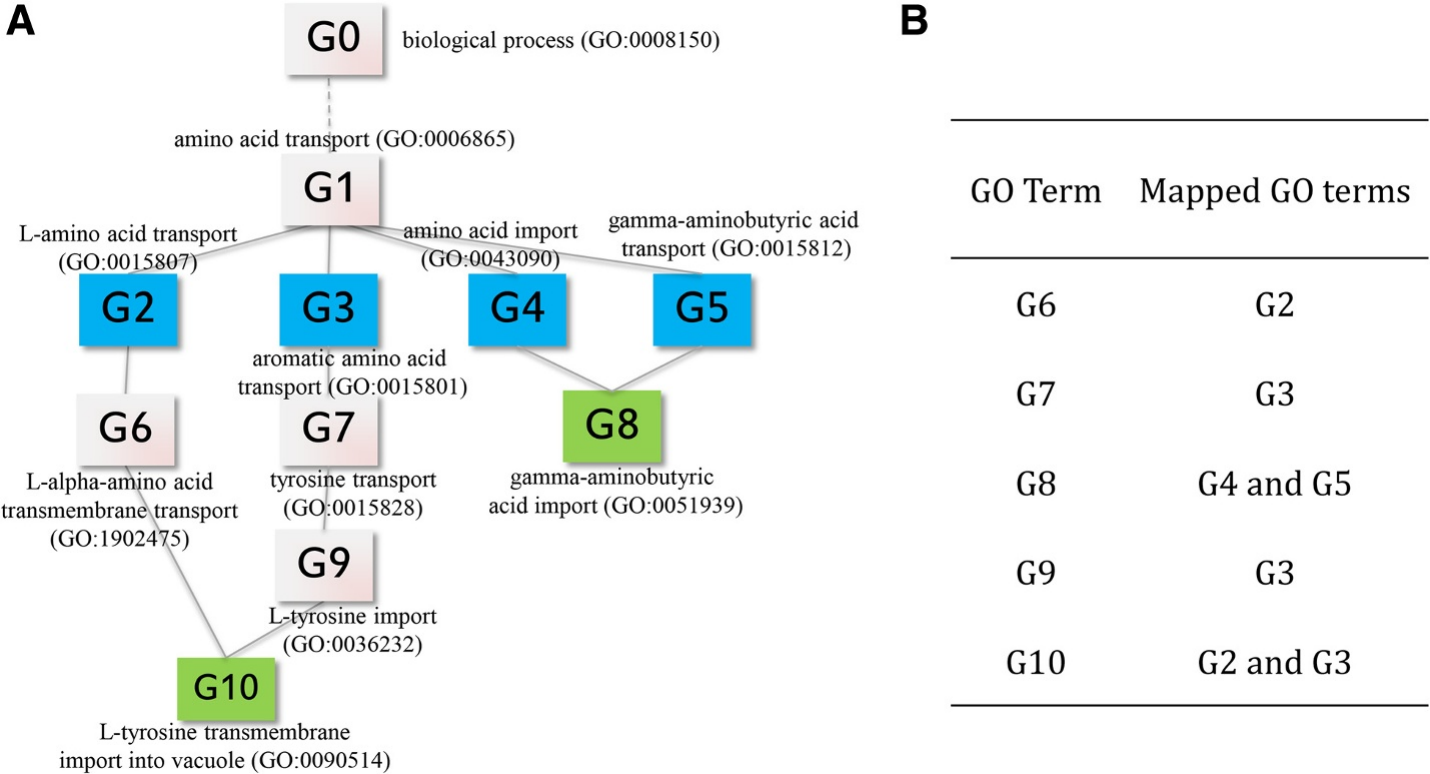
**Categorization based on the graphical structure of GO (GO Slim approach). A**. Section of the GO structure. GO terms of interest are colored in green and categories to which GO terms are assigned are colored in blue. In this example, each of the blue GO terms refers to a particular category. **B**. Mapping results by the GO Slim approach, which takes only the graphical structure of GO into account.

There are tailored cut-down versions of GO, GO Slims, to give a broad overview of the ontology content without the details [8], and there is also a script that automatically maps GO terms to GO Slim terms [9]. However, this script simply searches for ancestor GO terms that are also included in the GO Slim list and assumes that the GO graph has the architecture of a perfect tree in which all parent–child GO pairs are separated by the same distance. This is not true. Not all parent–child relationships in the hierarchical structure of GO have the same closeness in a biological sense. For instance, the relationship of ‘*protein serine/threonine kinase activity*’ (GO:0004674) and ‘*IkappaB kinase activity*’ (GO:0008384) is definitely much closer than that of ‘*biological process*’ (GO:0008150) and ‘*cellular process*’ (GO:0009987). Moreover, the GO architecture is more accurately represented by a directed acyclic graph, not a tree. Therefore, there can be more than one path from a GO term up to the root node and one GO term can be mapped to multiple GO Slim terms. As a result of the differences in parent–child closeness, a child term having two parent terms may be much closer to one of them in a biological sense. An example for such a case is shown in Figure 1A. According to the GO Slim mapping script, the term ‘*gamma-amminobutyric acid import*’ (G8) can belong to ‘*amino acid imports*’ (G4) and ‘*gamma-amminobutyric acid transport*’ (G5), since the graphical distances to both parent terms are identical (Figure 1B). However, the term ‘*gamma-amminobutyric acid import*’ is closer to ‘*gamma-amminobutyric acid transport*’ than ‘*amino acid imports*’ in the biological sense. Accordingly it is more reasonable to say that ‘*gamma-amminobutyric acid import*’ belongs to ‘*gamma-amminobutyric acid transport*’ , which can’t be deduced from the graphical distance alone. Hence, the biological closeness of GO terms, not the graphical distance, should be utilized for reliable GO analyses. Another problem with the tools mentioned before is that they assume independence of GO terms. Functions and processes encompassed by a specific GO term are a subset of the functions and processes encompassed by its parent term. Thus, GO terms cannot be independent but are associated. The use of redundant terms can lead to overestimation or underestimation of relevant biological processes or functions. Therefore, GO annotations should be analyzed in the context of the hierarchical structure of GO and by taking semantic distances between terms into account.

Several semantic similarity measures have been developed recently in order to approach some of these problems [10, 11]. As the name indicates, they provide a measure for how close two annotation terms are, and their calculation is based on the information content (IC), respectively the specificity of each annotation term. In the determination of the specificity of annotations, it is assumed that more frequently used terms are less specific [12–14]. Different types of semantic similarity measures have been introduced [10, 11, 15–17] and used in very diverse applications including the clustering of microarray data [18], the comparison of sets of genes and proteins from different species (GOTax) [19], the assessment of functional similarity of genes or proteins (G-SESAME) [20, 21] and the identification of new disease genes based on known disease annotations (MedSim, ACGR) [22, 23]. However, if one uses semantic similarity measures for the categorization of genes into groups of interest, one has to consider that categorization is not about predicting how close two terms are but assessing how well two terms go together.

To meet these demands, we developed Categorizer that assigns genes to pre-defined biological functions or processes based on their GO annotations. As biological functions or processes of interest are different from field to field, this new tool allows users to define their own categories.

## Implementation

Categorizer was implemented using a platform-independent language, Python, and thus it can run on any operating systems. For the user’s convenience, we also provide a pre-compiled version of Categorizer that runs on the Windows operating system.

**Figure 2:**
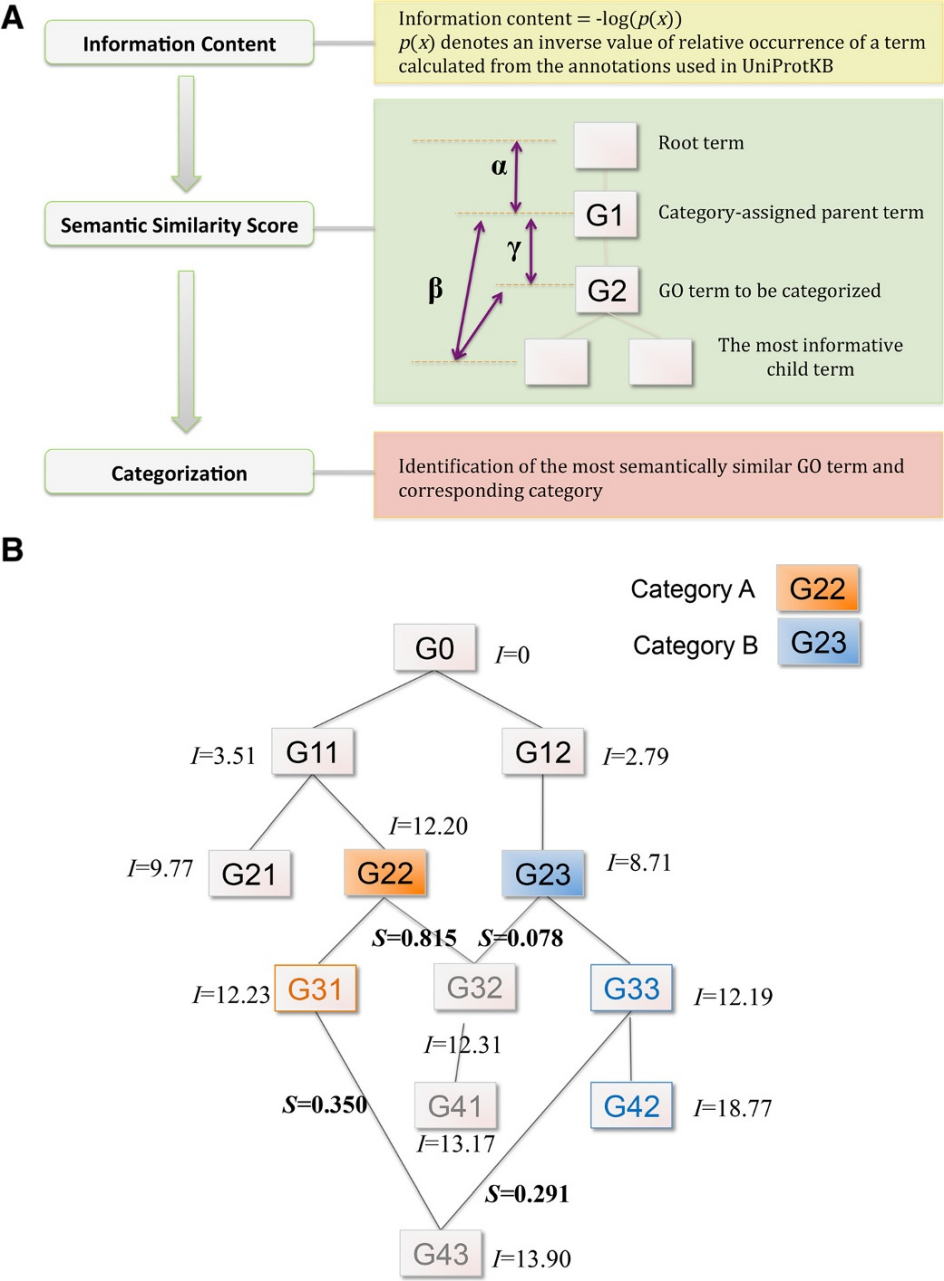
**Approach used in categorizer to assign genes to categories. A**. Three steps used in the categorization process: (i) *Information content* calculation, (ii) *semantic similarity score* calculations for parent–child pairs and (iii) *categorization* according to the semantic similarity scores. See the main text for details. **B**. Illustrative (synthetic) example for the calculation of semantic similarity scores. Information content scores (*I*) are shown for each GO term. G0 is a root term. In this example, a user defined two categories (*A* and *B*) and assigned G22 to category *A* (orange), and G23 to category *B* (blue). Semantic similarity scores (*S*) of several terms are also shown.

The overall scheme of the approach implemented in Categorizer is shown in Figure 2A. Categorizer employs three steps to categorize genes. (i) The IC scores of GO terms are calculated from the occurrence of GO annotations in UniProtKB-GOA [24]. This score denotes the biological relevance of a GO term, i.e., the more frequently a term is used the less relevant the term is. (ii) A semantic similarity score is calculated for all GO parent–child pairs based on their IC and the hierarchical structure in GO. (iii) According to the semantic similarity scores, genes with annotations are assigned to biologically appropriate categories. In the following sections, the details of these three steps are described.

### Information content (IC)

An IC score has to be assigned first to all GO terms in order to calculate semantic similarities. The IC represents the significance of GO terms in a biological sense. We assume that more frequently used GO terms are less significant [25]. We therefore counted all the occurrences of GO terms in a reference database. Here, we used all the proteins and their annotations in UniProtKB-GOA [24]. The hierarchical structure of GO was taken into account when counting occurrences. For example, when a protein has the annotation of ‘G21’ in Figure 2B, we also counted its parent terms, ‘G11’ and ‘G0’. When another protein has the annotation of ‘G22’ , we also increased the occurrences of ‘G11’ and ‘G0’. The overall occurrences in the given example are then ‘G0’ (+2), ‘G11’ (+2), ‘G21’ (+1) and ‘G22’ (+1). The occurrences are then divided by the number of annotations (which is two in the given example) in order to get occurrence probabilities of GO terms, *p*(x):


In this equation, orphan GO terms that have not been used in UniProtKB-GOA were assigned the lowest *p*(*x*) value of the terms, meaning the maximal IC. The probability is finally converted into an IC score that denotes the significances of each GO term:


In the given example, *I*(G0) is zero, which means that annotations with G0 are biologically meaningless. As the calculation of the IC score *I*(*x*) for all GO terms is computationally expensive, Categorizer comes with a file that contains pre-computed values. In addition, we also provide a script with which users can pre-compute other *I*(*x*) values taking their annotations of interest, e.g. UniProtKB-GOA (no IEA), Human GOA, or customized annotation files. Synthetic IC scores for each term in our example are shown in Figure 2B.

### Semantic similarity

When a specific GO term needs to be categorized, Categorizer searches for its parent terms that are assigned to a category and calculates semantic similarity scores with them. The semantic similarity scores are calculated as follows. IC scores are used to calculate (**α**) the semantic distance of a category-assigned parent GO term from the root node, (**β**) the semantic distance of the GO term to be categorized and its category-assigned parent term from their most informative child terms and (**γ**) the semantic distance between the category-assigned parent term and the GO term to be categorized (Figure 2A). All three scores are then combined in a final semantic similarity score [26]. Given two GO terms, for instance G32 (term to be categorized) and G22 (one of G32’s parent terms) (Figure 2B), we calculate the semantic similarity of these two terms as follows [26]:

#### Distance of a category-assigned parent GO term from the root node (α)

First, the semantic distance of the category-assigned parent term (G22) to the root term is calculated, which is defined as the difference in IC scores between the two GO terms (*x1* and *x2*):


where *r* is the root term and *p* is the parent.

Thus, the distance of G22 from the root term is *α* =12.20.

#### Distance from the most informative child terms (β)

Next, the average distance of the GO term to be categorized (G32) and its category-assigned parent term (G22) from their most informative child terms is calculated. The distance is defined as below:


where *c1* and *c2* denotes the most informative child node of *x1* and *x2*, respectively. If *c1* and/or *c2* do not exist, they are set to *x1* and *x2*, respectively.

In our example, G32 has the child term G41 and G22 has child the terms G31, G32, G41, and G43. The most informative child term of G32 is G41 and that of G22 is G43. Therefore, *β* = (*d*(*G*22, *G*43) + (*G*32, *G*41))/2 = ((13.90 - 12.20) + (13.17 - 12.31))/2 = 1.28.

#### Distance of a category-assigned parent GO term and a GO term to be determined (γ)

The third step is to calculate the distances between a category-assigned GO term (G22) and a GO term to be categorized (G32), which is defined as:


where *p* is a parent term assigned to a category and *x1* is a term to determine its category.

In this example, *γ* = *d*(*G*22, *G*32) = (12.31 - 12.20) = 0.11

#### Semantic similarity score

The final step is to calculate the semantic similarity score (*S*) from the three values, *α,* β, and γ:


where by 0 ≤ *S*(*x*1, *x*2) ≤ 1.

Consequently, the semantic similarity score between G22 and G32 is


### Categorization

Conventional semantic similarity measures were developed to assess how similar two GO terms are, but categorization is about assessing how well a specific term belongs to another term or a group of other terms. Thus, we use semantic similarity in the categorization process but require that a categorized term is a child of any term in the assigned category. For instance, two sibling terms, ‘DNA-templated transcription initiation (GO:0006352)’ and ‘DNA-templated transcription elongation (GO:0006354)’, are semantically very similar. They could be categorized to their parent term ‘RNA biosynthetic process (GO:0032774)’ because transcription initiation and elongation are both important steps in RNA biosynthesis. However, they cannot be categorized to each other because transcription initiation and elongation are two different molecular processes. Therefore, Categorizer first determines whether a term to be categorized is a child of only one or more category-assigned terms. If it is the child of only one term that has a category assignment, the similarity score of this parent–child pair is set to 1 and the term is assigned to the corresponding category. For a term that is a child of two or more category-assigned terms, Categorizer assesses semantic similarity between this term and all category-assigned terms and then assigns it to the category with the highest semantic similarity score. We demonstrate the procedure in the following examples:In the example shown in Figure 2B, the user assigned the term G22 to category A and the term G23 to category B. First, Categorizer automatically identifies child terms that belong to a single category only (e.g. G31 → A, G33 → B and G42 → B). For GO terms that have multiple parents, i.e. could belong to two or more categories (G32, G41, and G43), semantic similarity scores are calculated with the GO terms that are assigned to a category and their parents. Then the GO terms of interest are assigned to a category with the highest semantic similarity score.

#### Assignment example G32

Categorizer calculates pairwise semantic similarities of G32 with all the GO terms that belong to category A and are a parent of *G32*: *S*(*G22*,*G32*). In the same way, Categorizer also calculates semantic similarities of G32 with the terms in category B: *S*(*G23, G32*). Since *S*(*G22*,*G32*) = 0.815 and *S*(*G23*,*G32*) = 0.078, a gene with the annotation of G32 is more likely to belong to the category A.

#### Assignment example G41

Categorizer calculates the pairwise semantic similarities *S*(*G22*, *G41*) and *S*(*G23*, *G4*1). Since *S*(*G22*, *G41*) = 0.475 and *S*(*G23*, *G41*) = 0.071, a gene with the annotation of G41 should belong to the category A.

#### Assignment example G43

Categorizer calculates the pairwise semantic similarities *S*(*G31*, *G43*), *S*(*G22, G43*), *S*(*G23, G43*), and *S*(*G33*, *G43*). Since *S*(*G31*, *G43*) = 0.350, *S*(*G22*, *G43*) = 0.346, *S*(*G33, G43*) = 0.291 and *S*(*G23*, *G43*) = 0.064, we can infer that the term *G43* is closer to *G31* than *G33* in a biological sense and accordingly a gene with the annotation of G43 should belong to the category A.

One can allow a GO term to go into multiple categories if its semantic similarity score is above a user-defined threshold. For instance, a gene with the annotation of G32 can belong to category A and/or B depending on the semantic similarities and the user-defined threshold. The default threshold is set at 0.3 in Categorizer. This threshold value was determined by calculating an average semantic similarity score for two randomly selected GO terms that are linked directly or indirectly in a parent and child relationship. The average score was 0.10 ± 0.12 and accordingly Categorizer uses 0.3 as a default cutoff value for reliable categorization. After assignment of genes to one or several categories, enrichments of the categories are calculated.

### Enrichment analysis

Most GO enrichment analysis tools use simple statistical methods, including hypergeometric distribution, chi-square, Fisher’s exact test, and binomial probability [2]. When these methods are used to assess enrichment of categories, it is assumed that categories are independent. However, one gene may belong to two or more categories, and thus some categories may co-occur more frequently than others. Recently, a random model-based statistical enrichment analysis has been proposed [27]. Following this suggestion, Categorizer first calculates the probabilities of each category in a reference gene set:


where *p*(*c*) denotes a probability of category c in a reference gene set, *N* denotes the number of genes in a reference set, *M* denotes the number of categories, and *f*_*i*_(*c*) is 1 if the gene *i* is assigned into the category *c*, otherwise, 0. Then, the genes in the reference set are randomly assigned to categories according to the category probability, *p*(*c*), while retaining the number of assigned categories to each gene in order to keep the degree of categories. *L* different genes are randomly chosen from the reference, where *L* denotes the number of screened genes or genes of interest. The frequency of each category is then counted. These randomizations are repeated 1,000 times to obtain an average frequency and standard deviation of each category. With these averages and standard deviations, z-scores for each category are calculated as below:


The μ(c) and σ(c) denote an average number and standard deviation of category *c* obtained from the randomization. The *p*-values for each category are calculated from the *z*-scores.

### Utilization

**Figure 3:**
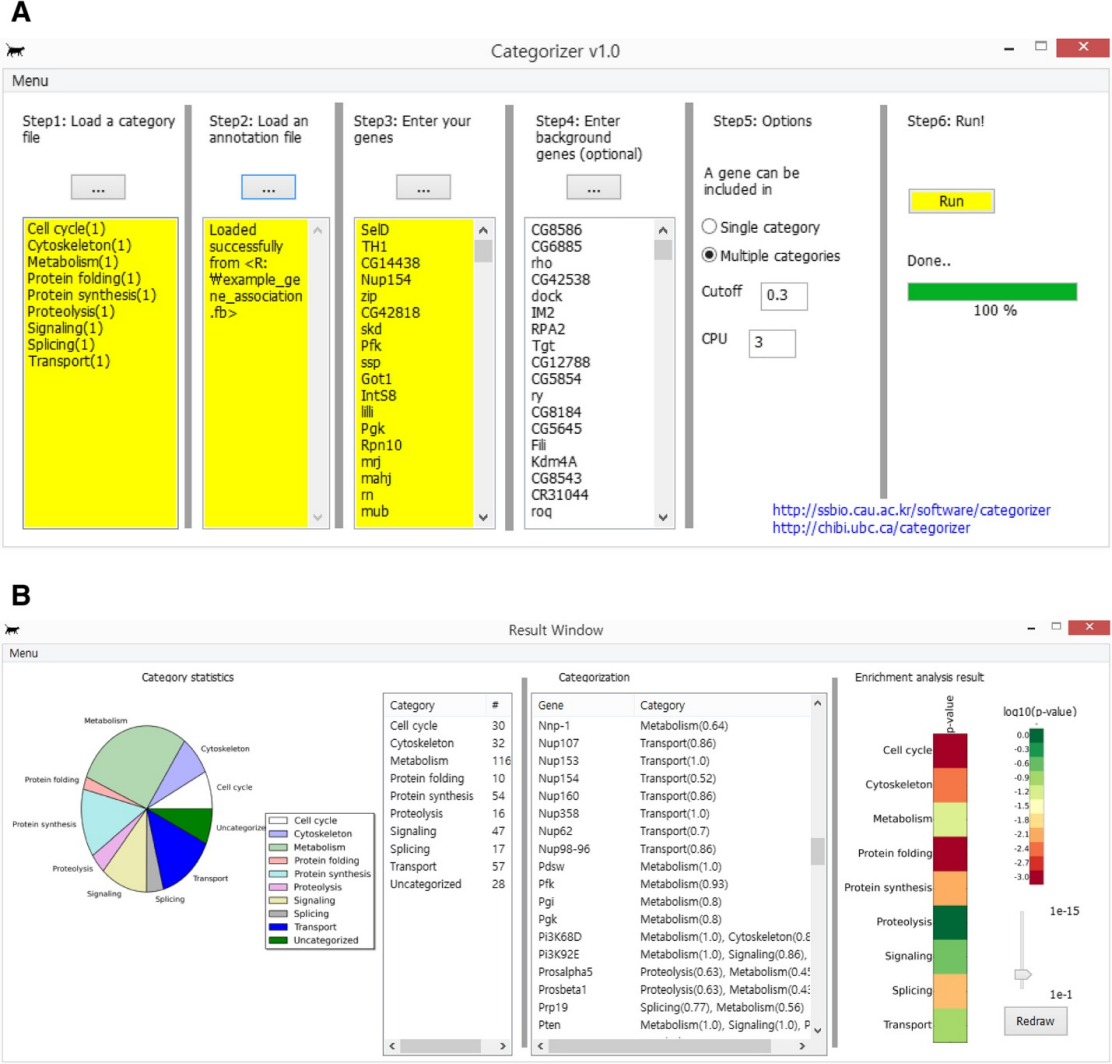
**Snapshots of the GUI of** ***Categorizer*** **. A**. Initial window for setting up the categorization parameters: category definitions, gene annotations, gene test set, background genes, and categorization options. **B**. Categorization results: category statistics (left), detailed categorization results (middle), and enrichment analysis result (right).

For practical categorization, the following key steps are carried out. First, a user has to define categories that are of interest and assign key GO terms to each category; a category is defined as a set of one or more GO terms. The user does not need to assign all GO terms to newly defined categories because Categorizer is capable of identifying semantically close GO terms and, by doing so, decides whether a GO term belongs to a category or not. Alternatively, the user can select among three commonly used category sets that are shipped with Categorizer: *biological processes*, *cellular localizations*, and *enzyme functions* (Table 1). For instance, the "*biological processes*" set contains 27 sub-categories. To run the software, at least three files (marked in yellow in Figure 3A) should be provided: (i) a category file defining categories and their GO terms, (ii) an annotation file containing gene-to-GO annotations, and (iii) a gene file containing the list of genes to be analyzed. A background gene file has to be provided for the category enrichment analysis.

**Table 1:** **Categories provided with categorizer**

Groups	Categories
Biological processes	Cell cycle, Cytoskeleton, Metabolism, Transcription, Translation, Protein folding, Proteolysis, Signaling, RNA processing, Splicing, Transmembrane transport, Intracellular localization, Protein transport, Nuclear transport, Vesicles, Golgi/ER, Mitochondria, Endo- and exo-cytosis, Lysosome, Peroxisome, Ribosomes, Phagocytosis/phagosome, Autophagy, Apoptosis, DNA repair, DNA replication, Receptors
Cellular localization	Cytoplasm, Mitochondria, Golgi, Nucleus, Cytoskeleton, Vesicle/Lysosome, ER, Extracellular
Enzyme functions	Hydrolase, Isomerase, Ligase, Lyase, Oxidoreductase, Transferase

## Results and discussion

### Genetic modifiers of Huntington’s disease

In order to demonstrate the functionality of Categorizer, we first analyzed the enrichment of specific categories in a set of genes that have been identified as genetic modifiers in *Drosophila* models of Huntington’s disease (HD). The data was compiled from NeuroGeM, a database of genetic modifiers of neurodegenerative diseases including HD, Alzheimer’s, Parkinson’s, Amyotrophic lateral sclerosis, and several Spinocerebellar ataxia types [28, 29]. Modifiers are genes that are capable of modulating disease phenotypes; in this case the neuronal cell death caused by protein aggregation.

We categorized genetic modifiers into 9 groups that are of interest to researchers studying HD: *cell cycle* (cell cycle, GO:0007049), *cytoskeleton* (cytoskeleton organization, GO:0007010), *metabolism* (metabolic process*,* GO:0008152), *protein synthesis* (*gene expression*, GO:0010467), *protein folding* (*protein folding*, GO:0006457), *proteolysis* (proteolysis, GO:0006508), *signaling* (signal transduction, GO:0007165), *splicing* (RNA splicing, GO:0008380), and *transport* (transport, GO:0006810). We loaded the *Drosophila* gene-to-GO annotation file (downloaded from FlyBase in March 2014), and entered the list of high-confidence genetic modifiers of HD (210 genes) obtained from NeuroGeM. As a reference, we entered all *Drosophila* genes that had been tested experimentally as modifiers (7896 genes). We allowed a gene to be included into multiple categories with the default cutoff value of 0.3.

With this information, Categorizer assigned the genetic modifiers to the defined categories. As shown in Figure 3B, categorization results for each gene are reported in the middle of the graphical user interface (GUI), i.e., the categories that each gene is assigned to are listed together with the semantic similarity score in parenthesis. On the left side of the GUI, there is a pie chart that displays the category statistics. In this example, the *metabolism* category is the largest while the *protein folding* category is the smallest. On the right side of the GUI, category enrichment analysis results are shown (see *Enrichment analysis* in *Implementation for details*). Consistent with the knowledge on the importance of the protein folding machinery in the pathogenesis of neurodegenerative diseases [30, 31], the category of *protein folding* is highly enriched among genetic modifiers of HD, though they account for only a small portion of the genetic modifiers. Additionally, the categories of *cell cycle*, *cytoskeleton*, *protein synthesis* and *splicing* are also enriched among the genetic modifiers of HD. This finding is consistent with recent research data on neurodegeneration and HD in particular [32–39].

In the given example, we categorized genetic modifiers of HD into broad biological processes and calculated their enrichment. However, if a user is interested in signal transduction, one could define categories such as NK-kappaB cascade or TOR signaling. It is up to the user to decide how specific or broad the defined categories are.

### Comparison of analysis results generated with Categorizer and classical approaches using GO Slim terms

Categorizer is different from GO Slim-based methods in that it identifies biologically relevant categories by using both the graphical structure of GO and the semantic similarities between GO terms. Therefore, we decided to compare the performance of Categorizer with that of the classical methods using GO Slim. First, we assessed the accuracies of category assignment by comparing assignment results of Categorizer and the GO Slim approach for a gold standard set of genes. Second, we evaluated the quality of enrichment analyses by comparing the results of the two approaches for the 210 high-confidence genetic modifiers of HD (used in Figure 3).

**Figure 4:**
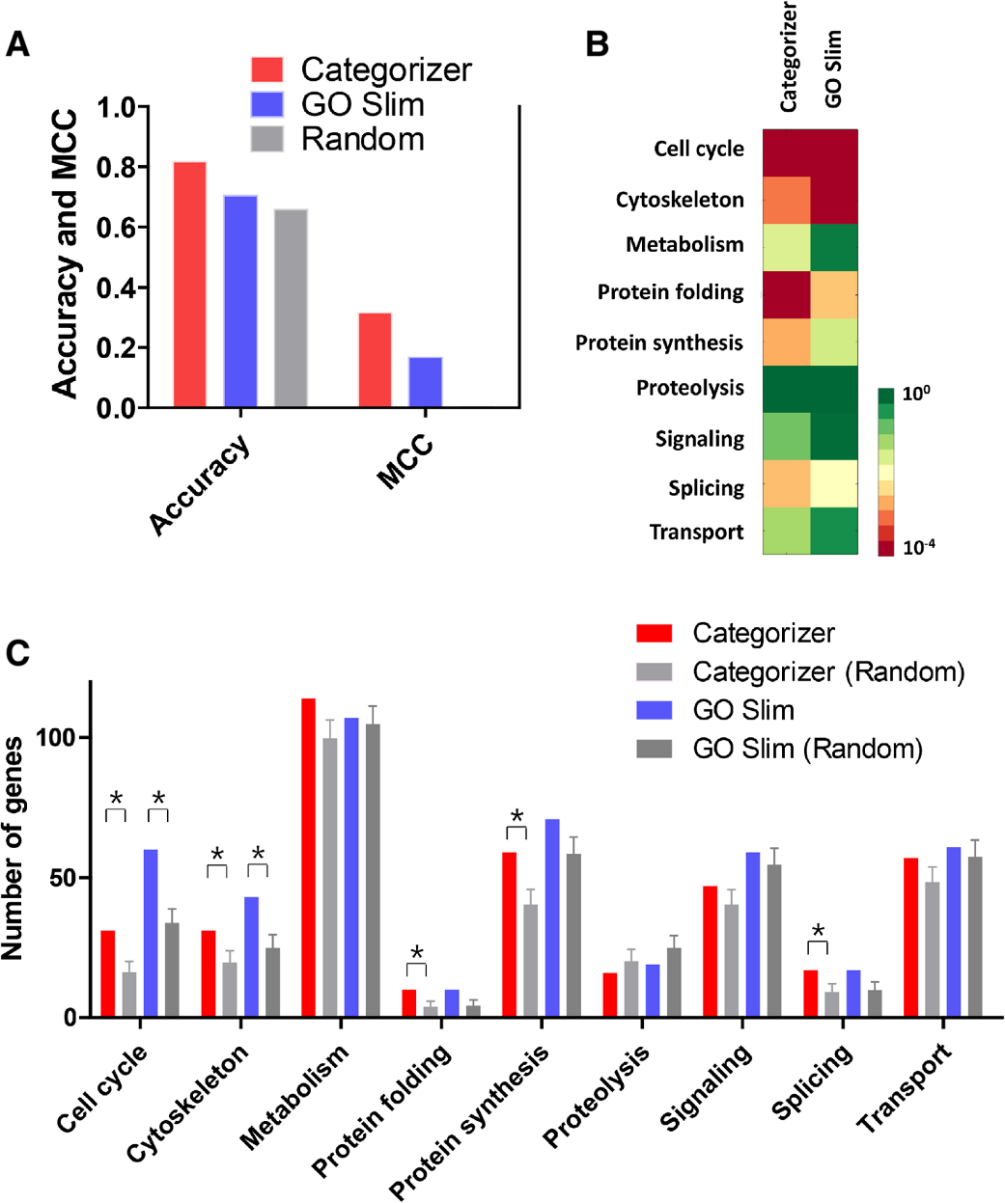
**Comparison of results generated by using Categorizer and a GO-Slim-based approach. A**. Overall accuracies of Categorizer, GO Slim and a random predictor. The categories of genetic modifiers obtained from a high-throughput screening study (Zhang et al.) were used as a gold standard. **B**. Enrichment of the 9 categories. All the genes tested for HD were used as a reference. **C**. Numbers of genes for each category in the test and randomized reference sets. Since we allowed multiple categorization, one gene may appear in several categories. Significantly enriched categories (*p* < 10^-2^) were marked as *.

Zhang et al. have previously categorized genetic modifiers, which they had identified in a high-throughput screen, manually into few broad biological processes (categories) based on the GO annotations of the modifiers [6]. We used these categorized genes as a gold standard to evaluate the accuracies of Categorizer and GO Slim-based methods. For this comparison, we customized a GO Slim ontology that is composed of the same nine GO terms that we used for Categorizer (see above). Then, *Drosophila* GO annotations were mapped to these nine terms with the help of the GO Slim assignment script *map2slim.pl*[9]. Categorization accuracy was calculated as below:


where *N* denotes the number of genes in the gold standard set; *N*_*C*_ denotes the number of total categories. As categorization is a multi-class problem, it is necessary to include both correct assignment to true categories and correct non-assignment to false categories when calculating accuracy. Briefly, we built two matrices named *G* and *P* denoting true answers and predictions, respectively. G(*g*, *c*) = 1 if a gene *g* in the gold standard set belongs to a category *c* and G(*g*, *c*) = 0 if the gene *g* does not belong to category *c*. P(*g*, *c*) = 1 if a gene *g* is categorized into a category *c* by Categorizer or the GO Slim-based method, respectively, and P(*g*, *c*) = 0 if the gene *g* is not categorized into category *c*. Thus, F(*g*, *c*) = 1 if G(*g*, *c*) = P(*g*, *c*) and 0 otherwise. The categorization accuracies of Categorizer and GO Slim are 81% and 70%, respectively (Figure 4A). As a control, a random predictor was built that randomly assigns genes to three categories. Three categories were chosen because the average number of assigned categories per gene in the gold standard by Categorizer and the GO Slim approach was 2.5. The accuracy of this random predictor is 65%. Since many genes are categorized into only a few categories, the number of correctly non-assigned genes has a big impact on this accuracy measure (hence the high accuracy of the random predictor). In order to deal with this issue, we also calculated the classical Mathew’s correlation coefficient (MCC) that is a suitable measure for evaluating unbalanced datasets. The MCC values of Categorizer, GO Slim-based method, and random predictor are 0.32, 0.17 and 0.0, respectively. Overall, these tests demonstrate that the category assignment of Categorizer is more accurate than a classical categorization with GO Slim terms and, thereby, underlines the importance of semantic similarity for this task.

Next, we compared the quality of enrichment analyses of the two approaches. We did so by analyzing the enrichment results of the two methods for the 210 genetic modifiers of HD used in Figure 3. The statistics of categories and enrichment results generated by using Categorizer and by the GO Slim-based approach are shown in Figure 4B and C. The GO Slim approach identified the categories ‘*cell cycle*’ and ‘*cytoskeleton*’ as significantly enriched among the genetic modifiers of HD, which is consistent with the results found by Categorizer (Figure 4B). However, the three categories of ‘*protein folding*’ , ‘*protein synthesis*’ and ‘*splicing*’ were not identified as enriched categories by the GO Slim approach (*p*-value > 10^-2^). This result of the GO Slim approach is in stark contrast to the literature on modifiers of neurodegenerative diseases, including HD. Genes whose products are involved in *protein folding, protein synthesis* and *splicing* are found in most screens for modifiers of neurodegenerative diseases that have been carried out to date [31, 40–42]. As shown in Figure 4C, both Categorizer and GO Slim assigned the same number of genes to the categories of *protein folding* and *splicing*. However, the GO Slim approach assigned more genes to these categories in the randomized model of the reference gene set than did Categorizer. Therefore, the *p*-values obtained by the GO Slim method were larger than those obtained by Categorizer. Interestingly, Categorizer identifies *protein synthesis* as enriched in contrast to the GO Slim approach, although Categorizer assigned fewer genes to the *protein synthesis* category than GO Slim. The solution to this conundrum is that Categorizer assigned much fewer genes in a reference set to *protein synthesis* than GO Slim. Overall, these comparisons reveal that Categorizer provides more reliable categorization and enrichment results compared to the conventional GO analysis method.

#### Comparison with other semantic similarity measures

**Figure 5:**
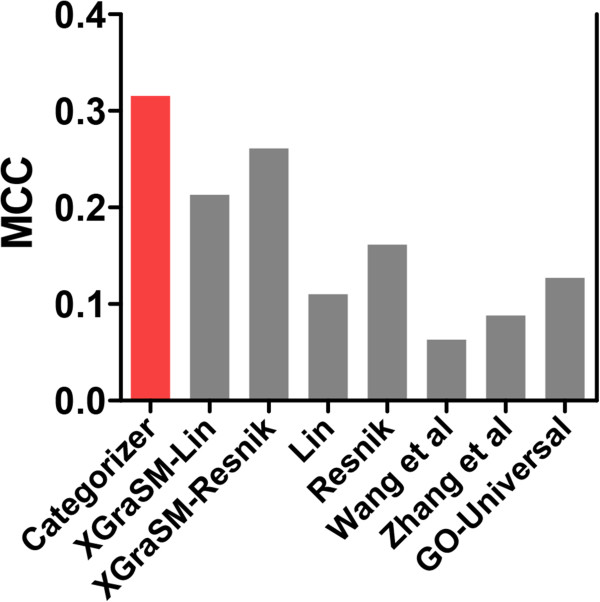
**Performance comparison of different semantic similarity measures and the one implemented in Categorizer.** MCC values were calculated with HD modifiers as done for the comparison of Categorizer and GO Slim in Figure 4A.

As different flavors of semantic similarity measures have been introduced [11], we assessed the accuracy of category assignment as a function of the semantic similarity measure. We used again as a gold standard the genetic modifiers of HD that were already categorized manually by experts in that field. Hence, we categorized HD modifiers based on different semantic similarity measures and assessed the accuracy of the categorization as we did for the GO Slim-based categorization in Figure 4A. We tested the semantic similarity measures developed by Lin, Resnik, Wang et al., and Zhang et al., as well as the one of XGraSM, and GO-Universal [11, 15, 43–45]. Cutoff values for each measure were determined, as for Categorizer, from the average similarity scores of randomly selected GO terms and measures were calculated using the annotations in UniProtKB including IEA. A key difference between these different metrics is the method used to calculate the IC. The approaches of Lin and Resnik use only the IC of the most informative common ancestor for similarity calculations, while XGraSM uses the averaged IC of all informative common ancestors (for details see [11, 15, 43–45]). The combined methods, XGraSM-Lin and XGraSM-Resnik, calculate semantic similarities based on Lin’s and Resnik’s semantic similarity metrics, but use the averaged IC of XGraSM. As shown in Figure 5, Categorizer outperforms all other measures commonly employed for assessing semantic similarity. Consistent with previous findings [11, 46], XGraSM provides the best categorization results of all the older methods (Figure 5). It is interesting to note that the MCC values calculated for XGraSM-Lin and XGraSM-Resnik are slightly higher than the MCC calculated for GO Slim. This finding provides further support for the importance of the semantic similarity in categorization.

## Conclusion

Here we developed a flexible and extendable tool that can be used to find over-represented categories within sets of genes. Categorizer classifies genes to categories according to biological meanings and assesses their enrichment. Thus, Categorizer offers a new way of enrichment analysis that allows focusing on processes that are of specific interest to the user.

## Availability and requirements

**Project name:** Categorizer

**Project home page:**http://chibi.ubc.ca/gsponer/categorizer

http://ssbio.cau.ac.kr/software/categorizer

**Operating system:** Platform independent

**Programming languages:** Python

**Other requirements:** None

**License:** Apache License 2.0

**Any restrictions to use by non-academics:** None
